# Non-Conventional Treatments for Conventional Chondrosarcoma

**DOI:** 10.3390/cancers12071962

**Published:** 2020-07-19

**Authors:** Varun Monga, Hariharasudan Mani, Angela Hirbe, Mohammed Milhem

**Affiliations:** 1 Division of Oncology, Department of Internal Medicine, University of Iowa, Iowa City, IA 52242, USA; varun-monga@uiowa.edu (V.M.); mohammed-milhem@uiowa.edu (M.M.); 2 Division of General Medicine, Department of Medicine, University of Iowa, Iowa City, IA 52242, USA; 3 Department of Medicine, Washington University, St. Louis, MO 63110, USA; hirbea@wustl.edu

**Keywords:** chondrosarcoma, isocitrate dehydrogenase, targeted therapy, chemotherapy, cyclin-dependent kinase inhibitors, immunotherapy

## Abstract

Chondrosarcomas are the most common malignant tumors of the cartilage, are seen predominantly in adults, and have varied clinical behavior. The majority of them affect the medullary canal of long bones and pelvic bones. The prognosis of chondrosarcoma is closely related to histological grading; however, the grading is subject to interobserver variability. Conventional chondrosarcomas are overall considered to be chemotherapy- and radiation-resistant, resulting in limited treatment options. The majority of advanced conventional chondrosarcomas are treated with chemotherapy without any survival benefit. Recent studies have evaluated molecular genetic findings which have improved the understanding of chondrosarcoma biology. Newer therapeutic targets are desperately needed. In this review article, we explore ongoing clinical trials evaluating novel ways of treating advanced conventional chondrosarcoma.

## 1. Introduction

Chondrosarcoma is a rare malignant tumor which forms cartilaginous matrix. The estimated overall incidence is 1 per 200,000/year and accounts for 20–30% of primary malignant bone tumors [1]. The majority of cases occur after age 50, with a slight male predominance [2]. The incidence of pediatric chondrosarcoma is less than 10% of all chondrosarcoma cases, with higher survival rates than adults [3,4]. Chondrosarcomas arising de novo are termed primary chondrosarcomas, while those developing in pre-existing benign cartilaginous tumors such as osteochondroma or enchondroma are referred to as secondary chondrosarcomas. Primary conventional chondrosarcomas constitute 85–90% of all chondrosarcomas and are further subdivided into central (centrally in bone), periosteal (surface of the bone or juxtacortical) and peripheral (cartilaginous cap of an osteochondroma) subgroups based on the site of origin within the bone unit [1] The most common sites are bones of the pelvis, mainly the ilium, followed by the diaphysis or metaphysis of the proximal femur and humerus, distal femur, and ribs. One percent of all chondrosarcomas arise in the small bones of the hands and feet. Nonconventional variants of chondrosarcoma include clear cell, mesenchymal and dedifferentiated chondrosarcoma and are not included in this review.

Tumors typically present with local swelling and/or pain of long-term duration. Cortical erosion or destruction is typically seen on a plain X-ray. Periosteal reaction is typically scant or absent. CT (contrast tomography) scan demonstrates matrix calcification. MRI (magnetic resonance imaging) can aid in the diagnosis of soft tissue extension of the tumor. The distinction between low-grade versus high-grade tumors can be made on dynamic contrast-enhancing MRI sequence, which aids in biopsy planning [5]. Histological grade is considered the single most important predictive marker of local recurrence or metastasis [6]. If two grades coexist in the same tumor, the area with the highest grade predicts prognosis. Other adverse prognostic factors include axial location of the primary tumor, size greater than 8 cm, metastatic disease at presentation, positive surgical margins and presence of pathological fracture [7,8,9]. The five-year survival rate for patients with grade 1 chondrosarcoma is approximately 83% and the major cause of death in these patients is from local recurrence typically involving the pelvis and shoulder girdle, which is difficult to manage surgically [10,11]. Grade 1 chondrosarcomas very rarely metastasize, and their primary treatment involves surgical intralesional curettage, burring, surgical adjuvant application of agents such as hydrogen peroxide or phenol and filling the cavity with bone graft [12]. About 5–10% of recurrent grade 1 tumors show transformation to a higher grade. Grades 2 and 3 chondrosarcomas have poor prognosis, with a five-year survival rate of this combined group of 53% [10]. Localized grade 2 and 3 tumors are surgically managed with wide en bloc resection [10]. Approximately 70% of patients with grade 3 tumors will develop metastatic disease, most often to the lungs, and less frequently to regional lymph nodes and the liver. 

Chemotherapy is generally not effective in conventional chondrosarcoma and there is no standard systemic therapy for advanced conventional chondrosarcoma. Multiple reasons for chemo-resistance have been proposed: slow proliferation rate, expression of multidrug-resistance 1 gene P-glycoprotein resulting in resistance to doxorubicin in vitro, increased activity of antiapoptotic and prosurvival pathways as suggested by high expression of Bcl-2 family proteins [13], and large areas of extracellular matrix and poor vascularity resulting in poor access of the antineoplastic agents [14]. Given the rarity of these cancers, randomized clinical trials to evaluate efficacy of systemic therapy is challenging.

Current chemotherapy recommendations include cisplatin and doxorubicin, extrapolated from recommended osteosarcoma treatment regimens. Their use is based on retrospective studies [15,16] and one prospective study [17] consisting of a small number of patients. In these studies [15,16,17], patients treated with doxorubicin monotherapy had a mean progression-free survival (PFS) of 2.5 months and those treated with the combination of doxorubicin with cisplatin had a PFS of 3.6 months. Interestingly, treatment with antihormonal therapy such as aromatase inhibitors resulted in a mean PFS of 6.7 months [16]. In a recent study examining national cancer database clinical outcomes of 865 patients with chondrosarcoma, chemotherapy did not show an overall survival benefit [18]. Five-year overall survival for stage III patients was reported at 60.6% with chemotherapy and 58.6% without chemotherapy (*p* = 0.709). Five-year overall survival for stage IV patients was 28.2% with chemotherapy and 31.2% without chemotherapy (*p* = 0.366). This outlines an urgent need for more effective and novel treatment strategies for the management of this cancer. Ongoing clinical trials for chondrosarcoma are presented in Table 1.

## 2. Targeted Therapies

### 2.1. IDH Inhibitors

Isocitrate dehydrogenase (IDH) is an NADP+ dependent enzyme that collateralizes the oxidative decarboxylation of isocitrate to alpha ketoglutarate during the Krebs cycle. In one study, somatic mutations of IDH 1 and IDH 2 were identified in approximately 50% of patients with chondrosarcoma and cartilaginous tumors [19]. These mutations are considered to be an early event [20], and result in the accumulation of a putative oncometabolite, 2-hydroxyglutarate [21,22]. Widespread DNA hypermethylation and impaired cell differentiation was noted in an IDH 2 mutant-expressing murine model of mesenchymal progenitor cells [23]. These progenitor cells ultimately led to the formation of undifferentiated sarcomas when grown as xenografts. In a retrospective study of 80 chondrosarcoma patients [24], activating IDH 1/2 mutations were found in 34% of patients and conferred a 5-year overall survival rate of 64% compared with a five-year overall survival rate of 93% without these mutations. This survival rate was noted to be lower than rates reported in other studies which have reported higher rates of IDH 1/2 mutations ranging from 56% [19] to 59% [25]. These mutations were present in 21% of grade 1 chondrosarcoma patients, 39% of grade 2 chondrosarcoma patients and 44% in grade 3 chondrosarcoma patients [24]. Inhibition of mutated IDH 1 in tumor cells should lead to decreased 2-HG production, thereby restoring normal cellular differentiation, thus providing a therapeutic benefit. 

AG-120 or ivosidenib is a first-in-class oral inhibitor of mutant IDH 1 that has been studied in a phase 1 trial that included chondrosarcomas. The drug was reportedly well-tolerated in one study and showed encouraging clinical activity in the 21 patients with advanced chondrosarcoma enrolled, with a stable disease rate of 52% and a median PFS of 5.6 months [26]. The six-month PFS rate was 39.5%. Sixty-two percent of patients did not have dedifferentiated histology thereby suggesting that ivosidenib may be more effective in conventional chondrosarcoma. There were no dose-limiting toxicities reported. The most common treatment-emergent adverse events were grade 1 or 2. Overall, 12 patients experienced ≥ grade 3 adverse events; only one patient developed grade 3 hypophosphatemia. Grade 1 or 2 ECG QT prolongation was reported in 23.8% patients. Various escalating doses of the drug were evaluated; 500 mg orally once daily was deemed to be most efficacious. Levels of 2-hydroxyglutarate (2-HG) were measured both in plasma and tissue specimens when available. Maximal 2-HG inhibition in plasma was achieved in the first 28 days at the dose of 500 mg daily. Up to 98.6% reduction in 2-HG levels was reported in tumor specimens compared with baseline values. A phase 1 clinical trial using another novel oral IDH 1 inhibitor, FT 2102, in relapsed and refractory AML and MDS has demonstrated efficacy in this patient population [27], leading to further evaluation in other IDH 1 mutated solid tumors including chondrosarcomas (NCT03684811). 

R140 IDH 2 mutations have also been described in three very high-grade conventional chondrosarcomas [24]. This has a therapeutic implication as a specific IDH 2-R140 inhibitor, enasidenib, has been recently approved by the FDA for relapsed and refractory AML. Hence, this new targeted drug may potentially be used in IDH 2 R140-positive chondrosarcoma patients and is currently being explored in an ongoing phase 1/2 study in advanced solid tumors including chondrosarcoma (NCT01915498).

### 2.2. Angiogenesis Inhibitors

Pathologic neovascularization has been reported in cartilaginous tumors [28] and is associated with aggressive clinical manifestations with higher incidence of metastasis [29]. Multiple antiangiogenic drugs in the form of tyrosine kinase inhibitors and fully humanized monoclonal antibodies targeting VEGF binding or the VEGF receptors, respectively, have been approved for clinical use in several cancer treatments. Pazopanib is an oral tyrosine kinase inhibitor and has been shown to inhibit the VEGF pathway in mice with chondrosarcoma xenografts [30].

A retrospective study including 10 patients with advanced chondrosarcoma, of which seven had conventional chondrosarcoma, reported outcomes with the use of pazopanib and ramucirumab as an antiangiogenic therapy, either as compassionate therapy or on clinical trials [31]. No partial responses were documented, and seven patients achieved disease stabilization for over six months. One patient with conventional chondrosarcoma who received ramucirumab had stable disease for 23 months. Median PFS was 22.6 months and median overall survival was not reached. Hypertension and fatigue were reported as the most common side effects [31]. Pazopanib as single agent was evaluated in unresectable and metastatic conventional chondrosarcoma in 47 patients [32]. This study used disease control rate at 16 weeks as the primary end point and it was met in 43% of the patients. The median overall survival was 17.6 months, and one patient had partial response. Median PFS was 7.9 months. Hypertension (26%) and elevated alanine aminotransferase (9%) were the most notable grade 3 adverse events.

Regorafenib is another oral multi-kinase inhibitor targeting tumor angiogenesis and the microenvironment. It has shown efficacy in patients with refractory colorectal carcinomas as well as gastrointestinal stromal tumors and is currently approved for these malignancies. A recent randomized, double-blinded, placebo-controlled phase 2 study included four parallel independent cohorts studying the efficacy of regorafenib as a single agent in patients with osteosarcoma, Ewing sarcoma, chondrosarcoma and chordoma [33]. Results of the osteosarcoma cohort demonstrated 65% of patients with stable disease at eight weeks compared with none in the placebo group. Treatment-related serious adverse events were reported in 13 patients and the most common side effects included chest pain, hypertension, hand-foot skin reaction, fatigue, and hypophosphatemia. No treatment-related deaths occurred. The results of the chondrosarcoma cohort are currently not yet available.

### 2.3. Cyclin-Dependent Kinase (CDK) Inhibitors

CDK inhibitors are widely used in the treatment of advanced breast cancer and have shown activity in lipomatous soft tissue sarcomas [34]. One clinical study evaluated CDK 4 expression in chondrosarcoma patient tissue samples and the levels of expression were associated with metastasis and recurrence of chondrosarcoma [35]. Treatment with palbociclib led to attenuation of CDK 4, thereby inhibiting chondrosarcoma cell viability via regulation of the CDK 4/RB signaling pathway. This suggests a role for CDK as a potential therapeutic target which can be evaluated in clinical trials. Abemaciclib is being explored in a phase 2 clinical trial in advanced soft tissue and bone sarcomas including chondrosarcomas (NCT04040205). In the MONARCH-3 trial, abemaciclib was used as a single agent in advanced breast cancer [36]. To date, the most notable side effect reported is diarrhea, occurring in 72.8% patients and mostly grade 1 or 2. Grade 3 or 4 adverse events included neutropenia (23.8%), diarrhea (9.5%), leukopenia (8.6%), and alanine transaminase elevation (6.4%).

### 2.4. Tyrosine Kinase Inhibitors and the Mechanistic Target of Rapamycin (mTOR) Pathway

A phase 2 trial of imatinib, a PDGFR/C-KIT tyrosine kinase inhibitor, and dasatinib, a tyrosine kinase inhibitor, failed to demonstrate clinically meaningful activity even though preclinical studies appeared to be promising [37,38]. Van Oosterwijk et al. reported that treatment with dasatinib resulted in successful sensitization for doxorubicin treatment in *TP53* mutant chondrosarcoma cell lines; thus, its use could be explored in combination with chemotherapy [39].

Phosphorylation of S6, a downstream marker for mTOR activity, has been shown to have increased activity in up to 69% of conventional and 44% of dedifferentiated chondrosarcoma [40]. Additionally, dual PI3K/mTOR inhibitor BEZ235 inhibited mouse xenograft chondrosarcoma cell lines JJ012 in vitro and in vivo, prompting more interest in the mTOR pathway in chondrosarcoma treatment [40]. One study investigating everolimus and/or doxorubicin showed no synergistic effect, but everolimus alone did have a suppressive effect on the tumor [41]. Sirolimus, given in combination with cyclophosphamide, showed a median PFS of 13.4 in a study of 10 unresectable chondrosarcoma patients [42]. One patient had an objective response and six patients had disease stabilization for at least six months. Grades 3–4 adverse events were observed in four patients with lymphopenia. Other common side effects included asthenia (70%), anemia (60%), nausea (60%), stomatitis (50%), and skin rash (50%). These studies suggest that mTOR inhibitors in combination with chemotherapy may stabilize disease but careful consideration of side effects is warranted. Other side effects of mTOR inhibitors include metabolic disorders such as hyperglycemia, hyperlipidemia, hypertension which should be considered.

### 2.5. Osteoclast Inhibitors

Tumor microenvironment elements, particularly osteoclasts, have been shown to influence the growth of chondrosarcoma in preclinical studies [43,44]. Using the swarm rat chondrosarcoma model, Otero et al. showed that zoledronic acid prevented cortical destruction, inhibited trabecular resorption, and resulted in decreased tumor volume in bone [44]. To evaluate zoledronic acid as a therapeutic target in humans, we are currently performing a phase 1B trial (NCT03173976) in patients with any grade chondrosarcoma with or without metastasis for whom resection of the primary tumor is indicated. Patients with dedifferentiated histology are permitted if they opt out of the standard of care doxorubicin-based regimen. Potential patients receive a standard dose of zoledronic acid three weeks preoperatively and another dose three weeks postoperatively. The primary objective of the trial is to compare osteoclast density in pretreated biopsy specimens versus post-treated resected tumor specimens. Local and distant relapse-free survival and overall survival are secondary endpoints.

## 3. Epigenetic Inhibitors

### 3.1. Hypomethylating Agents

As stated above, IDH mutations have been shown to create a hypermethylated phenotype in chondrosarcoma cell lines causing block in cell differentiation [45]. Inhibition of DNA-methyltransferase by decitabine in chondrosarcoma H-HEMC-SS cells led to restoration of genes essential for heparin sulphate (HS) expression in cells, causing decreased proliferation and tumor invasion properties [46]. Conversely, loss of DNA methylation in rat chondrosarcoma cells treated with decitabine showed increase in growth and invasiveness of the cancer cells. More research is needed to understand the epigenetic pathways [47] to determine whether hypomethylating agents such as 5-azacitidine or decitabine could have a role in chondrosarcoma treatment. We recently reported initial results of a phase 1B/2 clinical trial evaluating a novel combination of gemcitabine chemotherapy delivered at a standard fixed dose rate plus low-dose decitabine (0.1 or 0.2 mg/kg given subcutaneously twice weekly) in patients with soft tissue and bone sarcomas included patients with relapsed refractory chondrosarcomas (NCT02959164) [48]. Phase 1B results showed partial response in a patient with IDH-1 mutant chondrosarcoma and remained on study for seven months before discontinuing due to toxicity. Another patient with IDH wild type chondrosarcoma did not respond and had progressive disease after two cycles of protocol therapy. This suggests a response to the combination of chemotherapy and hypomethylating agent in IDH mutant chondrosarcoma. Final efficacy results and exploratory analyses are being compiled.

### 3.2. Histone Deacetylase Inhibitor

Preclinical studies have shown antitumor effects of histone deacetylase (HDAC) inhibitors on chondrosarcoma [49]. Modification of histone by acetylation is the key mechanism for regulation of gene expression, and various cancer types commonly have dysregulation of histone modification. HDAC inhibitors induce growth arrest and apoptosis in chondrosarcoma cells [49]. Currently, four HDAC inhibitors are approved for refractory T cell lymphoma and multiple myeloma. A phase 2 clinical trial of romidepsin in extra skeletal chondrosarcoma is completed and results are pending (NCT00112463).

The combination of the histone deacetylase inhibitor, SAHA (suberoylanilide hydroxamic acid), and the DNA hypomethylating agent decitabine was tested on chondrosarcoma IDH wild type, IDH1 mutant and IDH2 mutant cell lines in vitro and in vivo [50]. This resulted in decreased viability of all three chondrosarcoma cell lines and marked increase in the apoptosis marker, poly-ADP Ribose Polymerase (PARP). The combination of belinostat (HDAC inhibitor) is being explored with the longer-acting hypomethylating agent guadecitabine in a phase 2 clinical trial (NCT04340843). Romedepsin and belinostat cause gastrointestinal side effects such as nausea, vomiting, and anorexia, and have been the most commonly reported side effects to date (14% to 60%), along with anemia (37%), hyponatremia (8%), hypocalcemia (42%), anorexia (25%) [51].

## 4. Immune Checkpoint Inhibitors

Immunotherapy with immune checkpoint inhibition has become the cornerstone of therapy for numerous malignancies. Early phase clinical trials have shown some efficacy in soft tissue sarcoma subtypes [52]. This efficacy has been shown to be related to PD-L1 expression in some tumor types [53,54]. PD-1 is a cell surface protein receptor expressed on activated CD8+ T lymphocytes, B cells and natural killer (NK) cells. Blockage of the PD-1 pathway has produced favorable results in other cancers such as melanoma, non-small cell lung cancer and genitourinary cancers, and has become standard therapy for many malignancies [55].

About 41% of dedifferentiated chondrosarcomas show PD-L1 expression [56]. Despite promising preclinical work, clinical studies of immune checkpoint inhibitors in chondrosarcoma remain sparse. In the SARC028 clinical trial, one of five patients with dedifferentiated chondrosarcoma treated with pembrolizumab had an objective response [52]. A phase 1/2 trial of the combination pembrolizumab and doxorubicin in metastatic sarcoma is ongoing (NCT02888665).

In a phase 2 study evaluating the anti-PD1 antibody nivolumab, one patient with dedifferentiated chondrosarcoma showed partial response [57]. In another report, one patient with metastatic conventional chondrosarcoma treated with nivolumab had a favorable response [58]. There are several trials currently underway evaluating anti-PD1 antibodies in sarcomas including chondrosarcomas. A phase 1B dose escalation trial of nivolumab in combination with ABI-009 (nab-sirolimus) is currently ongoing in patients with bone and soft tissue sarcomas (NCT03190174). Initial phase 1 results showed that among nine patients treated, seven discontinued treatment, five due to progressive disease (PD), two due to grade 2 AE of acneiform rash and pruritus. Two patients have stable disease (SD) and are continuing treatment. Phase 2, enrolling 31 patients, is ongoing. Another phase 2 trial of nivolumab in combination with ipilimumab (a CTLA-4 inhibitor) is ongoing in patients with unresectable sarcomas including chondrosarcoma with mismatch repair deficiency (NCT02982486). A recombinant humanized PD-1 monoclonal antibody toripalimab that has conditional approval for use in the treatment of unresectable metastatic melanoma in China is currently being evaluated in a phase 1 trial across multiple tumor types including chondrosarcoma (NCT03474640). Reported severe side effects of immune check point inhibitors include colitis (11%), arthralgia (7%), rash (25%) [59].

Richert et al. described the immune landscape of chondrosarcoma from a cohort of 27 conventional and 49 dedifferentiated chondrosarcoma patient specimens [60]. Immunohistochemistry staining and molecular analyses showed PDL1 expression in 42.6% of patients. Colony stimulating factor 1 receptor (CSF-1R) was expressed by tumor associated macrophages in 89.7% of dedifferentiated chondrosarcoma and 62.9% in conventional chondrosarcoma. This suggests a role for immunomodulation of CSF-1R positive macrophages as a therapeutic target which could be explored in clinical trials.

## 5. Conclusions

Conventional chondrosarcomas largely remain resistant to conventional chemotherapy and the prognosis for advanced disease remains quite dismal. IDH-1 inhibitors are showing promising activity in IDH-1 mutant chondrosarcomas. Novel therapeutic targets and immunotherapy strategies are being explored for treatment of this rare disease.

## Figures and Tables

**Table 1:** Current clinical trials for conventional chondrosarcoma (March 2020).

Clinical Trial	Agent	Mechanism of Action	Study Design	Study Population	Status
**IDH Inhibitors**					
NCT03684811	FT-2102 or FT-2102 plus azacytidine	Oral IDH-1 inhibitor in combination with hypomethylating agent	Phase 1/2	Advanced solid tumors and gliomas including chondrosarcoma with IDH1 mutation	Recruiting
NCT04278781	AG-120	Oral IDH-1 inhibitor	Phase 2	IDH1 mutant chondrosarcoma	Recruiting
**Angiogenesis Inhibitors**					
NCT02389244	Regorafenib	Multi-kinase inhibitor, targeting antiandrogenic, stromal and oncogenic receptor tyrosine kinase	Phase 2	Metastatic bone sarcoma, chondrosarcoma	Recruiting
**Cyclin-dependent Kinase (CDK) Inhibitors**					
NCT04040205	Abemaciclib	CDK 4/6 inhibitor	Phase 2	Advanced bone sarcoma including chondrosarcoma	Recruiting
**P13k-Akt-mTOR Pathway**					
NCT02821507	Combination sirolimus and cyclophosphamide	mTOR inhibition with cyclophosphamide	Phase2	Metastatic or unresectable myxoid liposarcoma, chondrosarcoma	Recruiting
Osteoclast Inhibitors					
NCT03173976	Zoledronic acid	Bisphosphonate therapy influencing osteoclast activity	Phase 1b	Resectable chondrosarcoma	Recruiting
**Epigenetic therapy**					
NCT02959164	Combination gemicitabine and decitabine	Demethylation and inhibition of DNA synthesis	Phase 1B	Advanced malignancies, bone sarcomas	Active, not recruiting
NCT04340843	Combination belinostat and guadecitabine	HDAC inhibitor together with demethylation	Phase 2	Conventional Chondrosarcoma	Not recruiting
**Immune Checkpoint Inhibitors**					
NCT03190174	Combination nivolumab and nab-rapamycin (ABI-009)	Anti-PD1	Phase 1/2	Advanced malignancies, including sarcomas with deficient mismatch repair	Recruiting
NCT03474640	Toripalimab	Anti-PD1	Phase 1	Advanced malignancies including chondrosarcoma	Recruiting
NCT02982486	Combination nivolumab and ipilimumab	Anti-PD1	Phase 2	Unresectable sarcomas, including chondrosarcoma	Not yet recruiting
NCT02888665	Combination pembrolizumab and doxorubicin	Anti-PD1	Phase 1/2	Advanced sarcomas	Active, not recruiting
NCT03277924	Nivolumab plus Sunitinib	Anti PD-1 plus tyrosine kinase inhibitor	Phase 1/2	Advanced bone sarcomas	Recruiting
